# Let the soul speak: Promoting mental health awareness through arts and culture

**DOI:** 10.1192/j.eurpsy.2022.898

**Published:** 2022-09-01

**Authors:** S. Regev, R. Diler

**Affiliations:** 1 University of Haifa, Occupational Therapy, Haifa, Israel; 2 Nefashot, Nefashot, Jerusalem, Israel

**Keywords:** Belonging, Initiative, stigma, Activisem

## Abstract

**Introduction:**

Nefashot - (meaning “Souls” in Hebrew) is a social activist initiative that aims to promote mental health (MH) awareness in the public domain through cultural, artistic, and dialogue-based events.

**Objectives:**

The two main objectives of this project are: to raise awareness and promote dialogue about MH issues in the public sphere and to create an inclusive environment for people living with MH conditions where their voices can be heard.

**Methods:**

For this purpose, we have created a week of events around the *international mental health day* on October 10^th^. Our strategy for producing the MH week is by 4 stages: 1) Call for action which is published widely on social media 2) Collection of forms filled, connections and personal accompaniment 3) Event directed accompaniment, and group meeting around common topics 4) Publication as a group to strengthening the sense of belonging and enhancing community visibility.

**Results:**

Our 80 events over the last three years have been organized by creators, artists, people with and without mental illness, family members, and professionals. Participants are extended in the event production by geographical location, type of art or culture event, type of relation to MH (for example, family member) and by social groups (Arabic/English speakers, LGBT), as well as collaborations within the group.

**Conclusions:**

Promoting MH through public activism is ideal because it enable each participant to shape the process as well as the product. Furthermore, we find the relationship between art and MH enriching in both directions.

**Disclosure:**

No significant relationships.

